# Periodontitis and Parkinson's disease: a narrative review of associations, proposed mechanisms, and clinical considerations

**DOI:** 10.3389/froh.2026.1926663

**Published:** 2026-09-22

**Authors:** Jiayi Cheng, Xiaoyue Zhang, Jieyu Zhou, Li Liu, Jin Feng, Lei Zhao

**Affiliations:** 1 State Key Laboratory of Oral Diseases & National Center for Stomatology & National Clinical Research Center for Oral Diseases, West China Hospital of Stomatology, Sichuan University, Chengdu, Sichuan, China; 2 Department of Periodontics, West China School & Hospital of Stomatology, Sichuan University, Chengdu, China; 3 People's Hospital of Ningxia Hui Autonomous Region, Ningxia Medical University, Yinchuan, Ningxia, China; 4 Department of Geriatric Dentistry, West China Hospital of Stomatology, Sichuan University, Chengdu, China

**Keywords:** dysbiosis, neuroinflammation, oral health management, oral microbiome, Parkinson's disease, periodontitis

## Abstract

Periodontitis is a common chronic inflammatory disease, and oral health problems are frequent in people with Parkinson's disease (PD). Observational studies consistently indicate poorer periodontal status in PD, but evidence that periodontitis increases the incidence or progression of PD is inconsistent and does not establish causality. This narrative review summarizes current evidence on epidemiological associations, biologically plausible mechanisms, and dental management considerations. Human data support an association between PD and greater periodontal disease burden, plausibly related to motor impairment, reduced self-care capacity, salivary dysfunction, and altered oral microbial ecology. By contrast, proposed pathways from periodontitis to PD, including systemic inflammation, blood–brain barrier disruption, microbial products, and the oral–gut–brain axis, are supported mainly by preclinical, indirect, or low-level human evidence and should be regarded as hypotheses rather than established mechanisms. Observational reports of lower PD incidence among individuals receiving dental scaling do not demonstrate a preventive effect of periodontal treatment. In clinical practice, periodontal care should therefore be justified by established oral-health needs and individualized for PD-related motor, swallowing, cognitive, medication, and device-related considerations, rather than presented as a strategy to prevent or modify PD. Better longitudinal studies and interventional trials are needed to clarify temporality, mechanisms, and any neurological effects of periodontal treatment.

## 1 Background

Oral health is an important component of general health and quality of life. The World Health Organization estimates that oral diseases affect approximately 3.5 billion people worldwide. Severe periodontitis is among the most prevalent chronic conditions globally and has been estimated to affect about 10.8% of the population (1, 2). Because periodontal destruction is cumulative and can lead to tooth loss and impaired oral function, prevention, diagnosis, and long-term periodontal maintenance remain important across the life course (3). Multiple factors may contribute, including cumulative exposure to plaque-associated inflammation, comorbidity, polypharmacy, functional limitations, and socioeconomic barriers to dental care (4, 5). Periodontitis is also associated with several systemic conditions, although the strength and direction of these associations vary and causal mechanisms are not established for many of them (6–8). These observations provide a general context for considering periodontal health in populations affected by age-related neurological disease.

Parkinson's disease (PD) is a progressive neurodegenerative disorder in which age is one of the strongest established risk factors, with typical onset in later adulthood (9, 10). Age-related changes in mitochondrial function, genomic stability, proteostasis, and other cellular processes may increase vulnerability to neurodegeneration (11). Neuroinflammation is also an important component of PD pathobiology, although it is one element within a multifactorial disease process rather than a single established cause (12).

The literature on PD and periodontitis is heterogeneous and, importantly, the strength of evidence is not symmetrical in the two proposed directions. Human studies more consistently show that people with PD have poorer periodontal health, whereas evidence that periodontitis precedes or contributes causally to PD is inconsistent (13). Mechanistic studies provide biological plausibility but are dominated by animal, *in vitro*, post-mortem, or indirect evidence. The emphasis of this narrative review is therefore to distinguish established clinical observations from mechanistic hypotheses and to separate evidence-supported dental care from expert-derived clinical considerations. We synthesize three domains: (i) epidemiological associations between PD and periodontal health; (ii) proposed inflammatory, microbial, and oral–gut–brain pathways; and (iii) practical considerations for periodontal care in patients receiving PD pharmacotherapy or deep brain stimulation. This evidence-calibrated framing is intended to help clinicians interpret the literature without implying bidirectional causality.

## 2 Literature search and scope

For this narrative review, literature was identified through targeted searches of PubMed, Embase, the Cochrane Library, Scopus, and Web of Science for publications available through June 2026; ClinicalTrials.gov was also checked for relevant interventional studies. Search concepts included PD, periodontitis/periodontal disease, oral microbiome, inflammation, blood–brain barrier, and the gut–brain axis, and reference lists of relevant articles and reviews were examined for additional sources. Because the aim was a traditional narrative synthesis rather than an exhaustive systematic review, study selection was purposive and based on relevance to the review themes; no systematic-review-style workflow, formal risk-of-bias assessment, or certainty-of-evidence grading was undertaken.

To avoid conflating different levels of evidence, terminology throughout this review is calibrated to study design. Human observational findings are described as associations; animal and *in vitro* findings are presented as mechanistic hypotheses; findings from other neurodegenerative conditions are identified as indirect evidence; and clinical management points based primarily on expert opinion, case reports, or extrapolation are presented as considerations rather than formal guidelines. Accordingly, the absence of longitudinal or randomized evidence is treated as a major limitation when causal, preventive, or disease-modifying interpretations are discussed.

## 3 Evidence linking Parkinson's disease and periodontal health

### 3.1 Association of PD with poorer periodontal health

Human studies more consistently support an association between PD and poorer periodontal status than the reverse direction. Early reports found higher Community Periodontal Index of Treatment Needs scores and a greater prevalence of periodontitis among people with PD (14, 15). Subsequent cross-sectional studies reported greater gingival inflammation, bleeding on probing, plaque accumulation, probing depth, and clinical attachment loss in PD cohorts than in controls (16–20). The 2023 systematic review and meta-analysis by Chen et al. likewise found poorer periodontal parameters in PD but did not establish that PD causally increases the risk of periodontitis (13). More advanced PD has also been associated cross-sectionally with poorer oral or periodontal status (20). Overall, these human data support co-occurrence and a greater periodontal disease burden in PD, but the predominance of cross-sectional designs precludes firm causal inference.

A clinically plausible explanation is that PD-related functional impairment can make daily plaque control more difficult. Bradykinesia, rigidity, tremor, and other motor symptoms can reduce the precision and endurance required for toothbrushing and interdental cleaning, while cognitive or executive dysfunction may further impair self-care (21). These limitations provide a plausible pathway from PD to plaque accumulation and periodontal deterioration, but the magnitude of this contribution has not been quantified in longitudinal studies.

Salivary dysfunction may add to this burden. Patients with PD can experience both xerostomia and drooling; objective studies have often reported lower salivary flow, while drooling can coexist because of impaired oral motor control and swallowing (22, 23). Microbiome studies have also reported differences in salivary and subgingival communities in PD, including altered abundance of taxa such as *Prevotella*, *Eubacterium*, *Treponema*, *Actinomyces*, and *Fusobacterium* (24, 25). These findings are associative and cannot determine whether the microbial differences reflect PD itself, medications, oral-hygiene behavior, existing periodontitis, or other confounders. Likewise, higher gingival crevicular fluid concentrations of Interleukin-1β (IL-1β) together with a trend toward higher tumor necrosis factor-α (TNF-α), have been reported in PD cohorts (25). Together, these observations are compatible with a more inflammatory periodontal environment in PD, but they do not establish a PD-specific periodontal mechanism.

### 3.2 Association of periodontitis and oral-health indicators with PD incidence

Evidence that periodontitis precedes PD or increases PD incidence is less consistent. A population-based retrospective cohort reported a higher subsequent rate of PD among patients with periodontal inflammatory disease (26), whereas another nationwide retrospective cohort found only a weak or unclear association (27). Importantly, the 2023 meta-analysis found no statistically significant pooled association between periodontitis and incident PD in the available cohort or retrospective studies (13). A nested case–control study reported a lower observed incidence of PD among individuals who had received dental scaling (28), but this was an observational association rather than a treatment trial. Dental attendance, socioeconomic status, smoking, health behaviors, comorbidity, and other residual confounding may account for part of the association; the finding should therefore not be interpreted as evidence that scaling or periodontal therapy prevents PD. Similarly, tooth loss was associated with new-onset PD in a large cohort (29), but tooth loss is not specific to periodontitis and can reflect caries, smoking, access to care, comorbidity, and socioeconomic factors. Taken together, current epidemiology does not establish periodontitis as an independent causal risk factor for PD.

### 3.3 Proposed mechanistic links

Mechanistic studies provide biological plausibility, but much of the evidence derives from cell culture, animal models, post-mortem tissue, or other neurological diseases. Experimental *P. gingivalis* bacteremia has been shown to increase blood–brain barrier permeability through the Mfsd2a/Caveolin-1 transcytosis pathway (30), indicating one possible route by which periodontal infection or inflammation could influence barrier function. Rrginine gingipain A (RgpA) and other bacterial inflammatory products have been reported in systemic samples from PD cohorts (31), but systemic detection does not show that viable oral bacteria enter the PD brain. Trigeminal-nerve transport has been demonstrated for *P. gingivalis*-derived extracellular vesicles in an experimental cognitive-impairment model (32). Evidence of P. gingivalis DNA or gingipains in Alzheimer's disease brain tissue (33) is indirect with respect to PD and should not be treated as proof of the same route in PD.

A low-biomass sequencing study reported oral-associated bacterial DNA signatures in post-mortem brain regions from patients with PD, including the substantia nigra (34). However, post-mortem low-biomass microbiome studies are susceptible to contamination and cannot establish bacterial viability, timing, or route of entry. A 2024 study localized *P. gingivalis* gingipain antigens in substantia nigra neurons, but similar immunoreactivity was also present in neurologically normal control brains (35). These findings are therefore hypothesis-generating rather than disease-specific evidence. In experimental systems, bacterial products such as lipopolysaccharide can activate Toll-like receptor and NF-*κ*B-related inflammatory signaling in glial cells, providing a plausible mechanism by which microbial products could amplify neuroinflammation; whether this occurs at clinically meaningful levels in human PD remains unresolved (36). Systemic immune activation represents a second proposed pathway. Periodontitis can generate persistent local inflammation and episodic bacteremia, and experimental periodontal inflammation has been linked to circulating inflammatory mediators, blood–brain barrier changes, and neuroinflammatory responses (37, 38). In an *LRRK2* PD mouse model, oral *P. gingivalis* impaired gut permeability and altered peripheral immune responses (39), while IL-17A can exacerbate microglial activation and neurodegeneration in rodent PD models (40). These studies support biological plausibility for inflammatory amplification, but they do not establish periodontitis as a driver of human PD. An oral microbiome study also found that *P. gingivalis* Kgp type II was more common in PD with mild cognitive impairment and correlated with lower cognitive scores (41); this is an oral association and does not demonstrate adaptation, replication, or increased virulence of *P. gingivalis* within the central nervous system.

The oral–gut–brain axis is a third, more indirect hypothesis. The Braak hypothesis proposes that, in at least a subset of PD, pathological processes may involve the enteric nervous system and subsequently propagate along the vagus nerve (42). Periodontitis is accompanied by oral dysbiosis, and oral microorganisms are continuously swallowed with saliva (43, 44). Experimental studies and work in other inflammatory diseases suggest that some oral taxa can transiently persist in the intestine or alter gut microbial and immune conditions under permissive circumstances (45–49). This provides a conceptual basis for oral–gut interactions, but evidence that periodontitis-driven oral dysbiosis produces a reproducible gut microbial change that then initiates PD in humans is not established.

Animal studies add further mechanistic support but should not be extrapolated directly to human causation. Oral *P. gingivalis* exposure in an *LRRK2* PD mouse model was associated with impaired gut barrier function, altered immune responses, and increased *α*-synuclein-related changes in the enteric nervous system (39). Other oral bacteria, including *Veillonella parvula* and *Streptococcus mutans*, have been reported to worsen motor deficits and dopaminergic neuron loss in mouse models in the setting of oral inflammation (50). These findings support biological plausibility for an oral–gut–immune connection, but they do not demonstrate that periodontitis initiates or accelerates PD in humans.

In summary, the evidence hierarchy is uneven. The most direct human evidence concerns the association of PD with poorer periodontal health. By contrast, pathways from periodontitis to PD—including microbial translocation, systemic immune activation, blood–brain barrier disruption, and the oral–gut–brain axis (Figure 1)—remain mechanistic hypotheses supported mainly by preclinical, post-mortem, or indirect human data. These pathways may interact, but their relative contribution, temporal sequence, and clinical importance are unknown. Longitudinal human studies and carefully designed mechanistic investigations are needed before causal language is justified.

**Figure 1:**
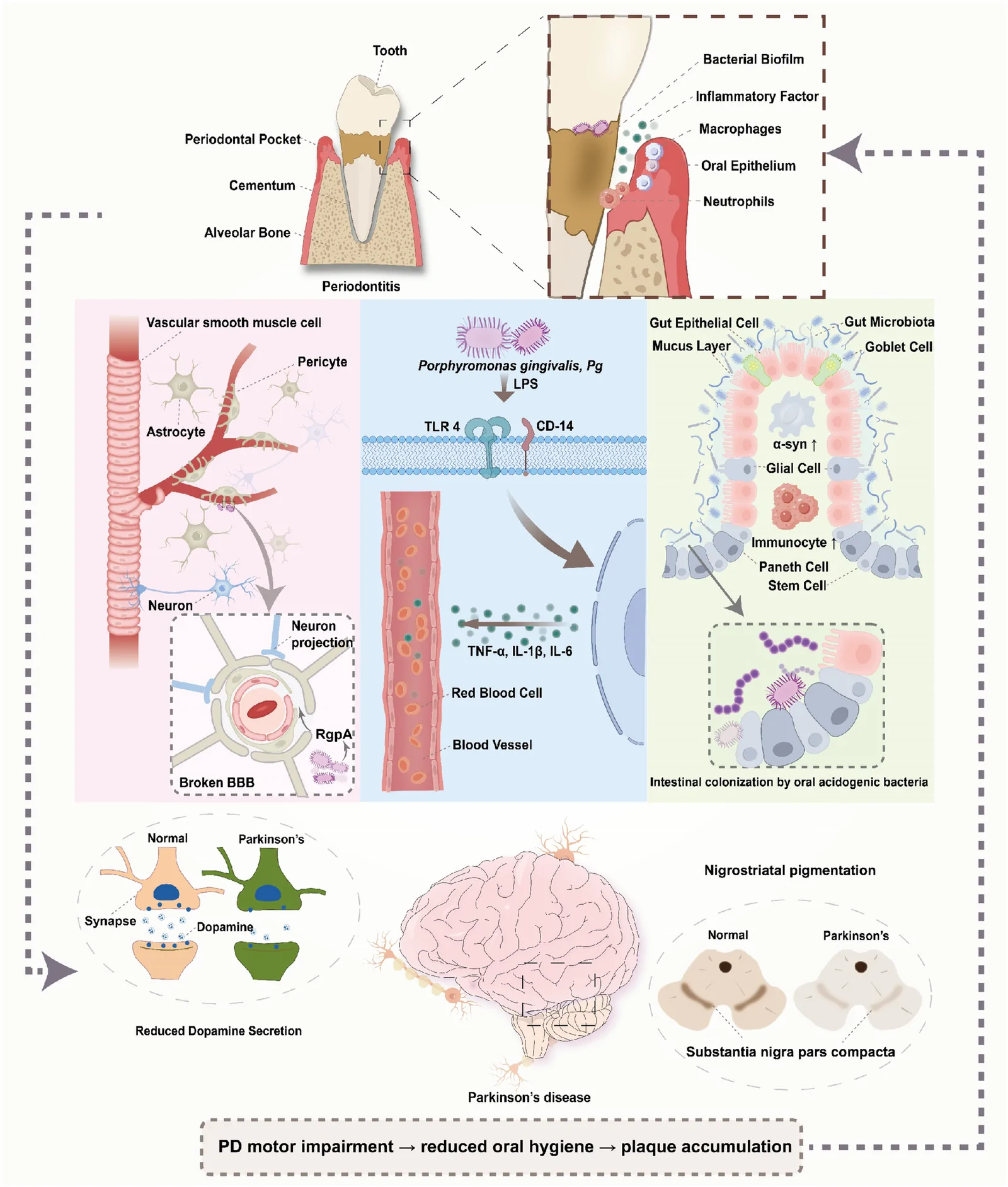
Conceptual model of clinical associations and hypothesized pathways linking periodontitis and PD. The pathways from periodontitis toward the central nervous system—blood–brain barrier disruption, systemic inflammatory signaling, and the oral–gut–brain axis—are mechanistic hypotheses supported mainly by preclinical, post-mortem, or indirect human evidence. The pathway from PD-related motor impairment to reduced oral hygiene and plaque accumulation is clinically plausible and consistent with observational data. Arrows indicate proposed directions of influence and do not denote proven bidirectional causality.

## 4 Clinical management considerations for patients with PD

The clinical points below are deliberately framed as considerations rather than formal guidelines. They are based on established dental principles, PD-related functional issues, observational studies, case reports, and expert or manufacturer guidance; PD-specific randomized evidence is sparse. Preventive oral care remains important because people with PD commonly experience plaque-control difficulties and periodontal disease (51, 52). Dysphagia and aspiration pneumonia are important complications of PD (53, 54), and reducing oral microbial burden is clinically reasonable. However, the literature reviewed does not establish that periodontal therapy specifically reduces aspiration pneumonia, mortality, PD incidence, or PD progression. Periodontal treatment should therefore be recommended for standard oral-health indications, with PD-specific adaptations used to improve safety, feasibility, and adherence.

Current PD treatments remain primarily symptomatic rather than curative (55). Dental planning should therefore focus on how PD manifestations and treatment influence the delivery of oral care, including motor fluctuations, autonomic symptoms, cognition, swallowing function, medication effects, and implanted devices. The following sections summarize practical issues that may warrant individualized modification of routine periodontal care.

### 4.1 Medication-related considerations

#### 4.1.1 Levodopa

Levodopa crosses the blood–brain barrier and is converted to dopamine, making it a cornerstone of symptomatic PD treatment (56). An *in vitro* study reported a mitogenic effect of L-dopa on human periodontal ligament fibroblasts (57), but no clinical periodontal benefit has been established. Gut bacterial tyrosine decarboxylases can reduce levodopa bioavailability before systemic absorption (58). By contrast, evidence that oral periodontal bacteria materially metabolize levodopa in patients is lacking; the presence of tyrosine-regulatory pathways in *P. gingivalis* (59) should not be interpreted as proof of levodopa metabolism. Accordingly, the main dental implications of levodopa are related to motor fluctuations, dyskinesia, orthostatic hypotension, nausea, and salivary symptoms rather than a proven direct effect on periodontal disease (56, 60).

Appointments can be planned during the patient's usual “on” period, when motor control is typically best, but a rigid universal interval after medication is not supported and should be individualized to the patient's regimen. Marked dyskinesia or tremor may justify shorter or staged appointments and additional positioning or stabilization support. Sedation or general anesthesia should not be treated as a routine response to involuntary movement; when contemplated, the indication should be individualized in light of dysphagia, airway risk, cognition, comorbidity, and drug interactions, with appropriate medical or anesthesia input. Assistance when changing chair position or standing may be prudent in patients with orthostatic symptoms.

#### 4.1.2 COMT inhibitors

Catechol-O-methyltransferase (COMT) inhibitors such as entacapone and tolcapone reduce levodopa metabolism and can prolong its therapeutic effect; tolcapone also has central COMT activity (61, 62). Direct adverse effects on periodontal tissues have not been established. Gastrointestinal adverse effects and dysgeusia may indirectly influence diet or oral-care routines, but these effects do not constitute evidence of a direct periodontal action.

Because COMT inhibition alters catecholamine metabolism (63), a cautious approach to vasoconstrictors in local anesthetics is reasonable in patients with cardiovascular instability or complex drug regimens. The literature reviewed does not provide PD-specific evidence for a universal maximum epinephrine dose. Clinicians should use the lowest effective vasoconstrictor dose, avoid intravascular injection, and monitor hemodynamic status when clinically indicated, with medical consultation for patients at higher cardiovascular or pharmacological risk.

#### 4.1.3 MAO-B inhibitors

Selegiline and rasagiline selectively inhibit monoamine oxidase B (MAO-B) at therapeutic doses and may be used alone in early PD or as adjuncts to levodopa in later disease (64). Xerostomia and dysgeusia can affect oral comfort and plaque control. Interaction risk depends on dose and concomitant medications; therefore, a complete medication review is more appropriate than a blanket prohibition of all dental vasoconstrictors. When there is uncertainty—particularly with higher doses, additional serotonergic or sympathomimetic drugs, or significant cardiovascular disease—coordination with the prescribing clinician is appropriate.

#### 4.1.4 General dental planning during pharmacotherapy

Rather than applying a fixed timing rule, appointments should be scheduled when the patient reports the most reliable motor control, often during a usual medication “on” period. Shorter visits, rest breaks, and staged treatment may be useful for patients with fatigue, fluctuating motor function, or difficulty remaining still. Medication lists should be reviewed at each visit because PD regimens and comorbid medications frequently change.

For patients with dysphagia or impaired oral motor control, a semi-upright or otherwise individualized chair position, effective suction, and isolation techniques may help reduce pooling of fluids when tolerated. These measures should be adapted to the patient rather than applied as universal requirements. Neurology consultation is not necessary for every routine dental visit, but may be useful for advanced disease, unstable motor fluctuations, complex drug interactions, uncertainty about decision-making capacity, or procedures requiring major treatment modification.

### 4.2 DBS therapy

Deep brain stimulation (DBS) is an established treatment for selected patients with moderate to advanced PD and can substantially improve motor symptoms (65). Evidence relevant to dental care, however, is sparse and consists largely of case reports and device-specific technical guidance. A single case report described an implantable pulse-generator pocket abscess in temporal association with apical periodontitis (66); this suggests a possible infection concern but cannot quantify risk or establish that routine periodontal procedures cause DBS infection. Another case report described uneventful dental treatment, including ultrasonic scaling, in a patient with DBS while noting manufacturer cautions about possible instrument–device interactions (67). These reports do not support universal statements that commonly used dental instruments will interfere with DBS.

Before treatment, clinicians should identify the presence of a DBS system, its manufacturer and model when possible, and the location of the pulse generator, and should review current device-specific instructions. When a dental device may generate electromagnetic interference, use should follow the manufacturer's recommendations and, if uncertainty remains, be discussed with the neurology or functional-neurosurgery team. Blanket avoidance of all powered dental instruments is not supported by the limited evidence. The same principle applies to other procedure-specific concerns: management should be device-specific rather than based on a single universal rule.

During treatment, monitoring should be proportionate to the patient's medical status and the planned procedure. Staging complex care, using manual instruments, or modifying appointment length can be considered when they improve comfort, safety, or cooperation, but these approaches are not mandatory for every patient with DBS. Figure 2 presents a conceptual clinical decision-making process for individualized care rather than a validated treatment algorithm.

**Figure 2:**
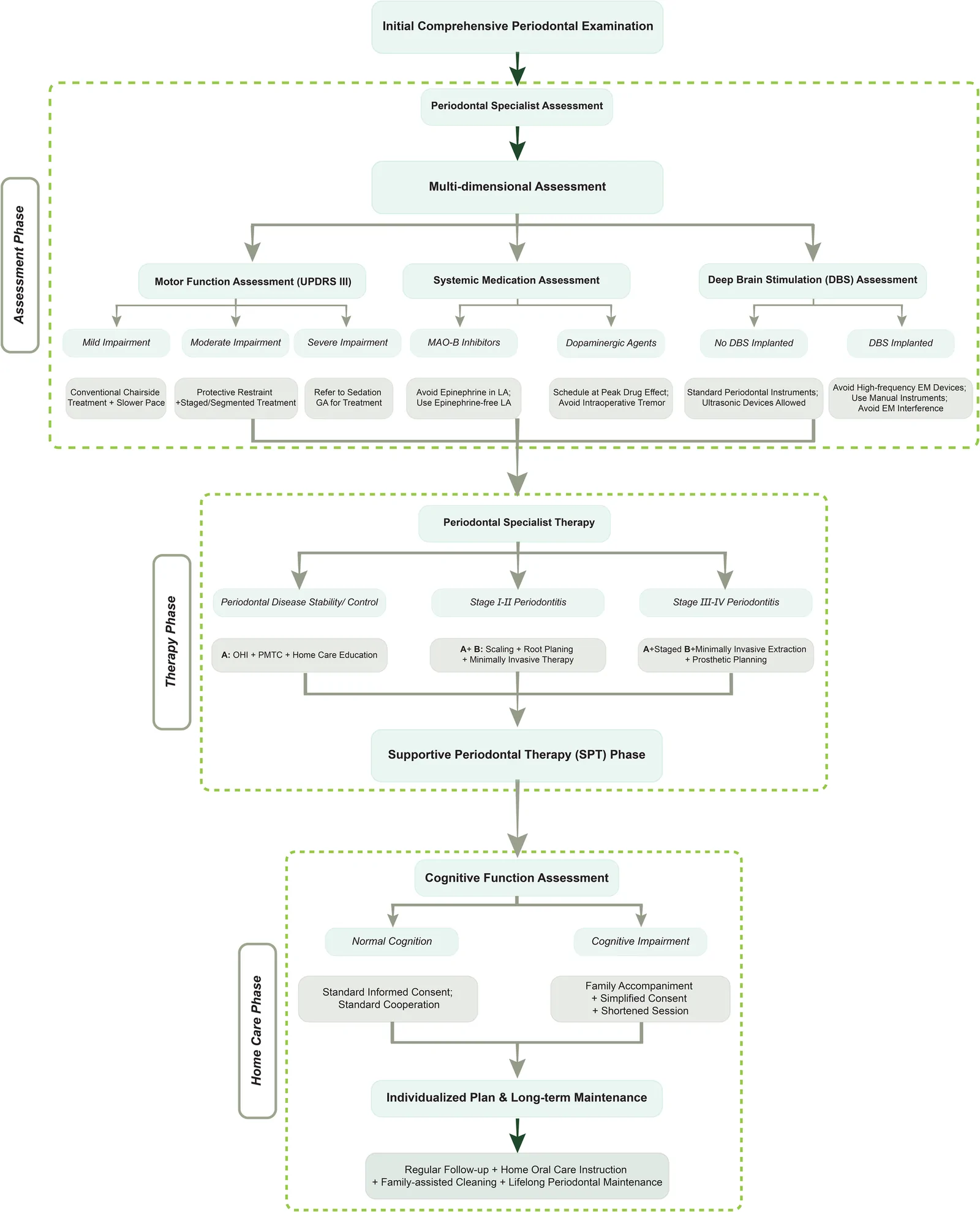
Conceptual flowchart for periodontal treatment decision-making in patients with PD.

## 5 Discussion

### 5.1 Limitations of current evidence and future research directions

The available evidence has substantial limitations that constrain causal inference. Most human studies are cross-sectional or retrospective, so they primarily capture co-occurrence rather than temporal sequence (13). Definitions of periodontitis vary across clinical examinations, self-report, and administrative coding, while PD ascertainment ranges from specialist diagnostic criteria to physician diagnosis or administrative diagnostic codes. This heterogeneity reduces comparability across studies and may contribute to inconsistent effect estimates. The long prodromal phase of PD also makes temporality difficult to establish, because periodontal exposure, oral-care behavior, medication use, comorbidities, and socioeconomic factors may change for years before a formal PD diagnosis (29). These issues are particularly important because the pooled epidemiological evidence has not established that periodontitis increases PD incidence (13).

This review itself also has methodological limitations. As a traditional narrative review, the search and study-selection process was not designed to be exhaustive, and no formal risk-of-bias assessment, meta-analysis, or formal certainty-of-evidence assessment was performed. The synthesis is therefore susceptible to selection and publication bias and should not be interpreted as a systematic estimate of effect. Future work should prioritize prospective cohorts with repeated, standardized periodontal and neurological assessments; studies that explicitly address smoking, healthcare utilization, socioeconomic status, medication exposure, and other time-varying confounders; and mechanistic investigations that distinguish viable organisms from microbial products or post-mortem signals. No randomized trial evaluating whether periodontal treatment changes PD motor, non-motor, biomarker, or progression outcomes was identified in the literature reviewed. Such trials would be required before periodontal therapy could be discussed as a PD-modifying intervention.

### 5.2 Clinical management considerations

Clinical implications should remain proportional to the evidence. Because PD can impair dexterity and self-care, powered toothbrushes, simplified home-care routines, and caregiver assistance may be considered when they improve plaque control and are acceptable to the patient (68). Interdental brushes, floss, and antimicrobial mouth rinses should be individualized to dexterity, swallowing safety, periodontal needs, and caregiver support rather than prescribed uniformly. Dental recall should also be risk-based; reviews of dental attendance in PD do not justify a universal twice-yearly minimum for every patient (69). Xerostomia is commonly reported and warrants assessment when symptomatic; saliva substitutes, sugar-free sialagogues, hydration strategies, and medication review may be considered when appropriate (23). A randomized study in care-dependent nursing-home residents—not specifically patients with PD—found benefits from monthly professional oral care and individualized instruction (70). This evidence is relevant by extrapolation to some care-dependent patients, but it does not support monthly professional cleaning for all people with PD. Interdisciplinary collaboration between dental and neurological teams may be useful when motor, cognitive, swallowing, medication, or device-related issues complicate routine care (71).

## 6 Conclusion

Current human evidence supports an association between PD and poorer periodontal health, but it does not establish a reciprocal causal relationship between PD and periodontitis. Evidence that periodontitis increases the incidence or progression of PD is inconsistent, and pooled observational data have not demonstrated a significant association with PD risk (13). Reports of lower PD incidence among people who received dental scaling are observational and should not be interpreted as evidence that periodontal treatment prevents PD. Proposed mechanisms involving microbial products, systemic inflammation, blood–brain barrier disruption, and the oral–gut–brain axis are biologically plausible but remain supported mainly by preclinical, post-mortem, or indirect evidence. Periodontal care remains important for established oral-health reasons and should be adapted to PD-related motor, swallowing, cognitive, medication, and device-related needs. At present, it should not be presented as a strategy to prevent or modify PD. Longitudinal studies and interventional trials are needed to clarify temporality, mechanism, and whether periodontal treatment has any neurological effects beyond its established oral benefits.
